# Machine learning-based prediction of celiac antibody seropositivity by biochemical test parameters

**DOI:** 10.1038/s41598-025-08225-6

**Published:** 2025-07-03

**Authors:** Signe Ulfbeck Schovsbo, Michael Charles Sachs, Margit Kriegbaum, Anne Ahrendt Bjerregaard, Line Tang Møllehave, Susanne Hansen, Bent Struer Lind, Tora Grauers Willadsen, Allan Linneberg, Christen Lykkegaard Andersen, Line Lund Kårhus

**Affiliations:** 1 Center for Clinical Research and Prevention, Copenhagen University Hospital – Bispebjerg and Frederiksberg, Copenhagen, Denmark; 2 Section of Biostatistics, Department of Public Health, University of Copenhagen, Copenhagen, Denmark; 3 Copenhagen Primary Care Laboratory (CopLab) Database, Research Unit for General Practice and Section of General Practice, Department of Public Health, University of Copenhagen, Copenhagen, Denmark; 4 Department of Clinical Biochemistry, Copenhagen University Hospital Hvidovre, Hvidovre, Denmark; 5 Department of Clinical Medicine, Faculty of Health and Medical Science, University of Copenhagen, Copenhagen C, Denmark; 6 Department of Hematology, Rigshospitalet, Copenhagen, Denmark

**Keywords:** Machine learning, Prediction, Celiac disease, Laboratory tests, Epidemiology, Coeliac disease, Gastrointestinal diseases, Nutrition disorders

## Abstract

The diagnostic delay in celiac disease (CD) is currently a burden for individual and society. Biochemical tests may be used in risk-identification of CD to reduce the diagnostic delay, and we aimed to explore prediction models for CD antibody seropositivity. We developed two prediction models in a cohort study using data from primary care in greater Copenhagen (2006–2015). All patients with CD antibody tests were included. Two candidate sets of predictors were considered: (1) all blood tests measured, (2) tests deemed clinically relevant pre-study or previously studied. Both models assessed test results 5 years before CD-testing. We developed and evaluated prediction models in 10-fold cross-validation framework for each set of predictors. Four machine learning methods were combined in stacked models using SuperLearner. 54,877 patients were included, 672 CD antibody seropositive. Cross-validated estimated area under the curves were 0.68 and 0.63. Distributions of predicted probabilities overlapped substantially between patients with CD antibody seropositivity and seronegativity. Food allergen antibody and IgA were the most important predictors. Biochemical tests had low predictive power but provided methodological insights for future models. These may improve by combining biochemical tests with other clinical information but should preferably aim to stay clinically implementable.

**Supplementary Information:**

The online version contains supplementary material available at 10.1038/s41598-025-08225-6.

## Introduction

Celiac disease (CD) is a lifelong autoimmune disease caused by an abnormal immune response triggered by the ingestion of gluten in genetically susceptible individuals^1^. CD has a strong human leucocyte (HLA) association^2^and the enzyme tissue transglutaminase (TTG) deamidates the gluten peptides, which are presented to HLA DQ2 and HLA DQ8-restricted CD4 + T-cells^2,3^. This stimulates an immune response with plasma cell production of autoantibodies immunoglobulin (Ig)A and IgG against the TTG enzyme, and today the detection of these antibodies is an important step in diagnosing CD. The only current treatment is a gluten-free diet. The disease affects approximately 1% of the population^4^ but up to 80% remain undiagnosed^5^. Diagnosed CD has been associated with increased risk of mortality^6^ and morbidity^7^. Classical symptoms include diarrhea, weight loss, and growth failure, but the disease may also present with subtle and unspecific findings, such as iron deficiency anemia, or symptoms like headaches, and fatigue^5^. This challenges the diagnostic process, and the diagnostic delay is currently estimated to be approximately 6 years on average, and many have been shown to have a delay of more than 10 years^8,9^.

The diagnostic delay may increase the individual and societal burden of disease, e.g. incident CD-patients had on average 8 days more lost work than comparators 5 years before diagnosis, and 13.7 days more 5 years after diagnosis^10^. Similarly, the delay may result in extensive healthcare utilization^11^, poorer quality of life^9^ and longer time until effect of treatment^12^. Due to the current hampered diagnostic process and long diagnostic delay, interest has been shown in predicting CD. One model^13^ assessed clinical indications of CD as predictors of undiagnosed CD in a cohort study. The model had a low predictive value with area under the receiver-operating characteristic curve (AUC) ranging from 0.49 to 0.53, which the authors attributed to the fact that the indications for testing included in the study did not characterize undiagnosed CD well. In small samples, one study^14^ showed good performance in predicting diagnosed CD patients by symptoms (216 patients) and ICD-9 codes (202 patients). Another model^15^ assessed both symptoms and risk factors as predictors with good discrimination in internal validation (c-statistics 0.82 in children, 0.76 in women and 0.80 in men), but performance decreased in external validation. The authors ascribe the decrease to lack of information on first-degree relatives in the external validation. As the symptoms of CD are commonly unspecific, usage of subjective symptoms and risk factors may be challenged when assessed as predictors of CD.

Therefore, objective measurements may prove more feasible as predictors. In this regard, genetic prediction models^16,17^ have been conducted with good performance, however, the clinical implementation of these is limited, as primary care does not readily have access to such test material at time of initiation of examination. Bicohemical tests may be another objective parameter used in prediction. Today, screening for CD is recommended in international guidelines as part of active case finding for unexplained iron-deficiency^18–21^, elevated liver function tests^18–21^, vitamin B12, or folate deficiency^20^ in adults, and in unexplained iron-deficiency, abnormal liver biochemistry or IgA-deficiencies in children^22^. In addition to the biochemical blood tests manifestations mentioned in the guidelines, vitamin D- and calcium deficiencies^23^ have also been identified in CD. Due to such known associations between biochemical abnormalities and CD, biochemical test results may act as important clinical predictors of future CD, and identifying patterns of biochemical variations as warning signs of CD may help reduce the diagnostic delay. This has only been done in one recent study, showing high accuracy (AUCs ranging from 0.77 to 0.86 depending on model) in predicting a group of selected highly seropositive cases among controls without suspicion of CD^24^. A Danish observational study from primary care, utilizing the Copenhagen Primary Care Laboratory (CopLab) database^25^ found CD antibody seropositivity to be associated with lower ferritin, hemoglobin, cobalamin, and folic acid levels, and higher transferrin, alanine aminotransferase, and alkaline phosphate compared with CD antibody seronegativity. The study only assessed selected associations. We aimed to expand on the findings from the CopLab study by utilizing the same database including all numeric test results among all test parameters available to explore data-driven prediction models to predict CD antibody seropositivity in patients tested for CD in primary care.

## Methods

### Source of data

We used data from the CopLab database^26^ and the Danish National Patient Register (NPR)^27^ to conduct this observational cohort study.

CopLab is based on data from the Copenhagen General Practitioners’ Laboratory (CGPL, Københavns Praktiserende Lægers Laboratorium). The laboratory was founded in 1922 and was the only laboratory serving general practitioners and practicing specialist in the former county of Copenhagen and municipality of Copenhagen from 2000 until its closure in 2015. CopLab was established in 2015 to make data available for research. CopLab therefore contains results of all tests performed and collected in CGPL from July 1, 2000, to December 31, 2015, from approximately 1.3 million individuals. The database contains 112 million results of biochemical parameters ranging from blood, semen, and urine, but also clinical physiological tests, and various cardiac and lung function tests. The CopLab database is administered by the Research Unit for General Practice and Section of General Practice, Department of Public Health at the University of Copenhagen.

All Danish citizens are registered with a unique civil registration number in the Danish Civil Registration system^28^ which enables individual-level linkage between the CopLab database and the Danish nationwide registers. NPR is administered by the Danish Health Authority and contains recorded administrative and clinical information on all hospital contacts in Denmark from 1977 and onwards (outpatient care contacts, emergency room contacts and psychiatric contacts were included from 1995). In NPR, individual diagnoses are registered according to the World Health Organization’s International Classification of Diseases, 10th edition (ICD-10)^29^ (until 1994 the previous 8th edition (ICD-8) was used). Data from CopLab and the NPR was merged by Statistics Denmark using the civil registration number and all analyses were made in the Computing environment of Statistics Denmark.

### Study population

CD antibodies were measured at CGPL from 2006 to 2015, and all patients with at least one numeric result of CD antibodies measured at CGPL in this period were included (*N* = 58,101). We applied no restriction based on area of residence, number of tests performed or type of clinical setting.

**Fig. 1:**
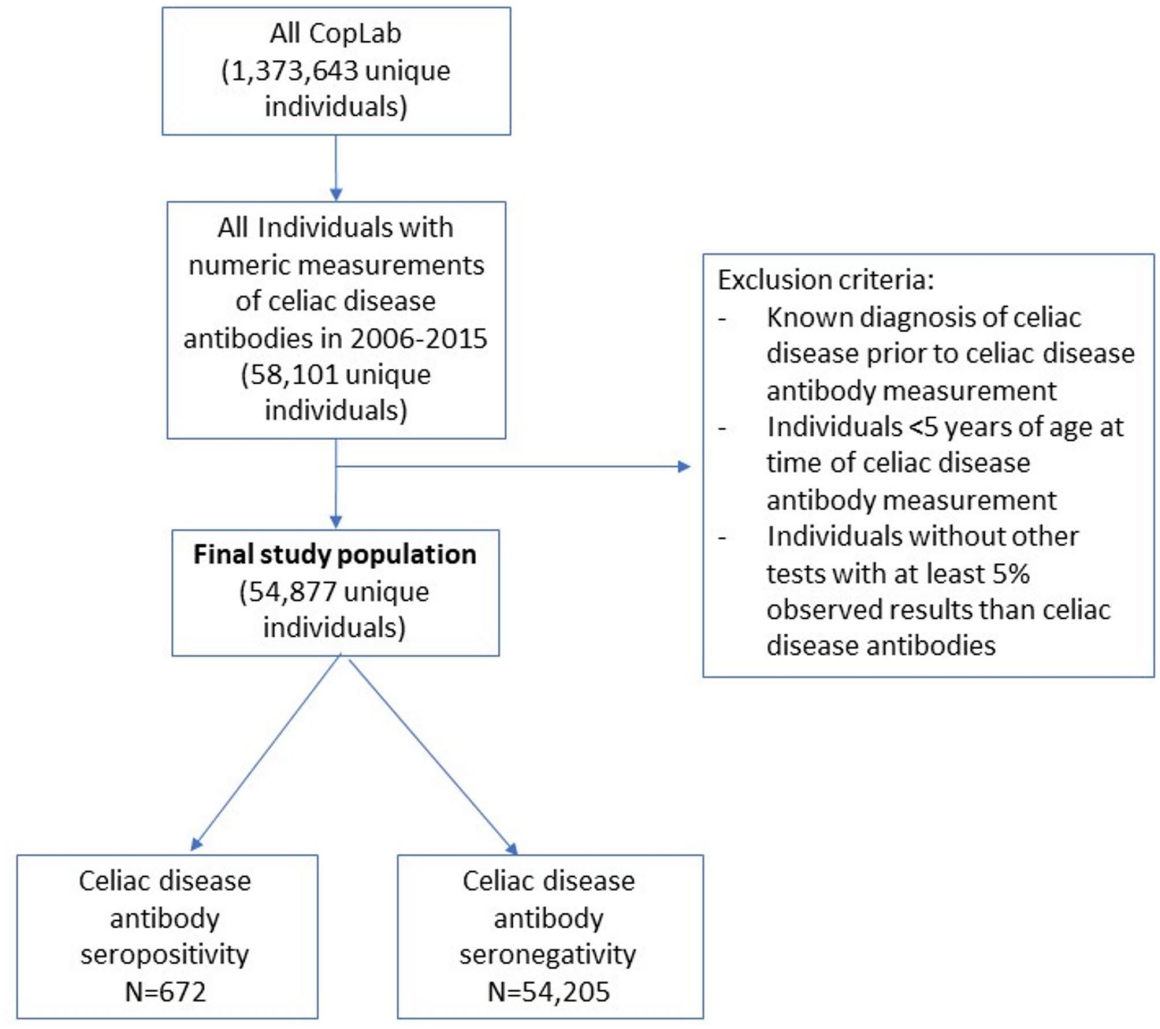
Flowchart, description of the selection of the study population.

Diagnosed CD was identified as ICD-8 code 269.00 or ICD-10 code K90.0 in NPR. Patients who had a registered CD diagnosis in NPR before the CD antibody test, were excluded. To ensure equal follow up time, patients < five years of age at the time of antibody test, and patients without tests with at least 5% observed results other than CD antibodies, were excluded. The final study population consisted of 54,877 unique patients (Fig. 1).

### Outcome

CD antibody serology was measured as described in detail by Kårhus et al.^25^. The outcome was binary, and a patient was considered CD antibody seropositive if TTG-IgA ≥ 7 kU/L, TTG-IgG ≥ 7 kU/L, deamidated gliadin peptide (DGP)-IgA ≥ 10 kU/L, or DGP-IgG ≥ 10 kU/L. In case of multiple CD antibody tests in the same patient (on different time points), we included only the first positive result or the last negative result to ensure only one outcome-observation per patient.

### Predictors

For tests performed at CGPL, many of the analytical methods have previously been described, and the comparability of test parameters over time have been ensured and documented. All other test parameters were assumed comparable over time if the test reference intervals were unchanged over time. Numeric results and results with the signs < or > were included.

### Candidate sets of predictors

Two approaches were used resulting in two candidate sets of predictors before model development was initiated: (1) a data-driven approach with exclusion criteria kept minimal and objective (termed the full list), and (2) a clinical approach with selection based on clinical relevance pre study or the previous CopLab-study^25^ (termed the curated list). Please refer to supplementary data for details (Supplementary Table S1 and S2):

1. Full list of test parameters: All blood test parameters measured at CGPL during a period of five years before CD antibody measurement (*n* = 271) were included. A total of 164 test parameters were excluded due to the following criteria: (1) no longer being used in clinical practice at time of model development (*n* = 2), (2) unknown measurement methods (*n* = 3), (3) conversions of other results already included (*n* = 3), and (4) less than 1000 requisitions (*n* = 156).
2. Curated list of test parameters: Blood test parameters measured at CGPL during a period of five years before CD antibody measurement deemed clinically relevant pre study or previously assessed^25^ were included (*n* = 17). Selected blood test parameters (*n* = 4) performed at an external laboratory were also included due to clinical relevance without restrictions on number of requisitions. Clinical relevance was evaluated through clinical consensus basis.

Afterwards, test parameters that were missing in 95% or more of the total study population were excluded in both the full list (*n* = 32) and in the curated list (*n* = 5). After this exclusion based on missingness, all predictors of the curated list were contained in the full list. Sex and age at time of CD antibody testing were included as predictors in both lists.

**Fig. 2:**
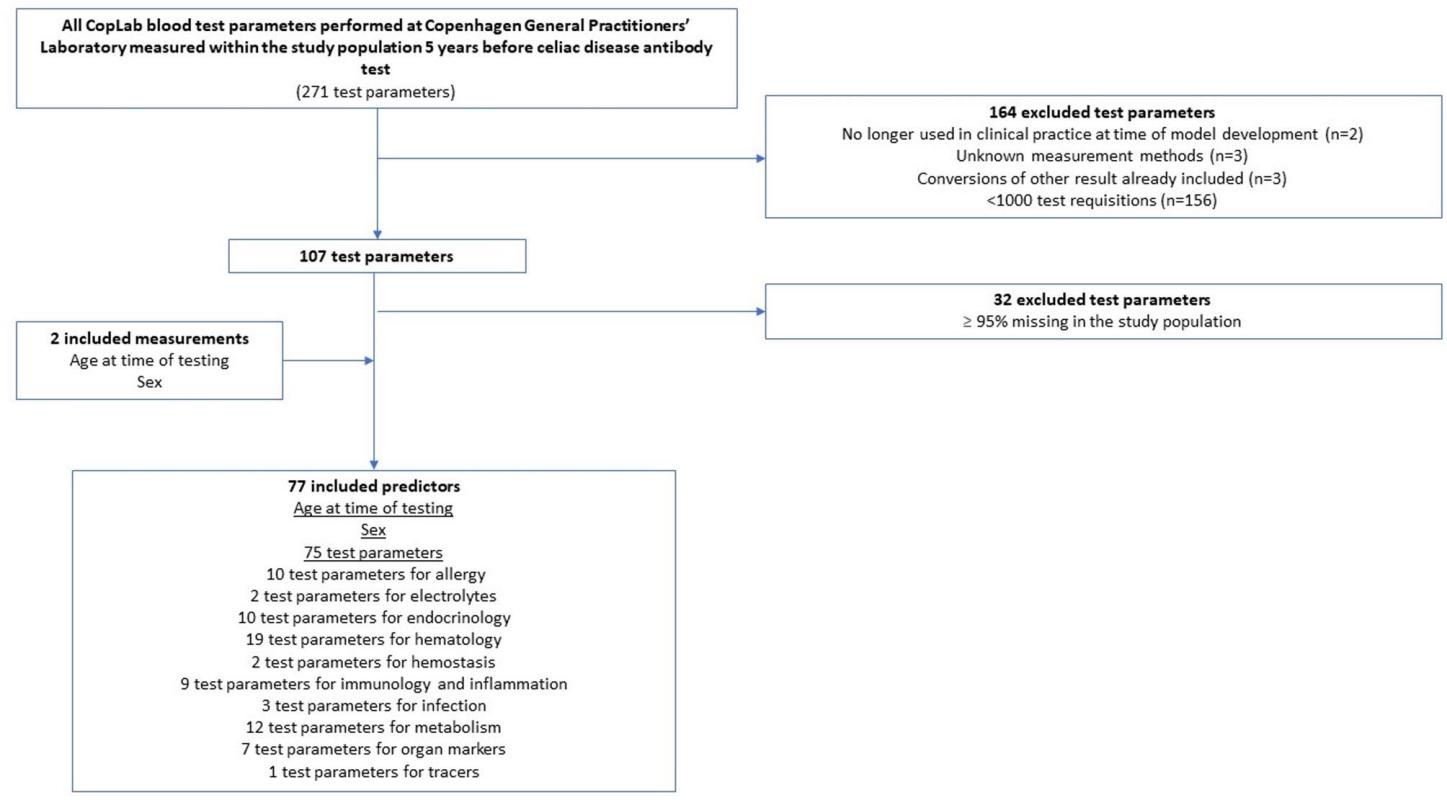
Candidate set of predictors: Full list of test parameters.

**Fig. 3:**
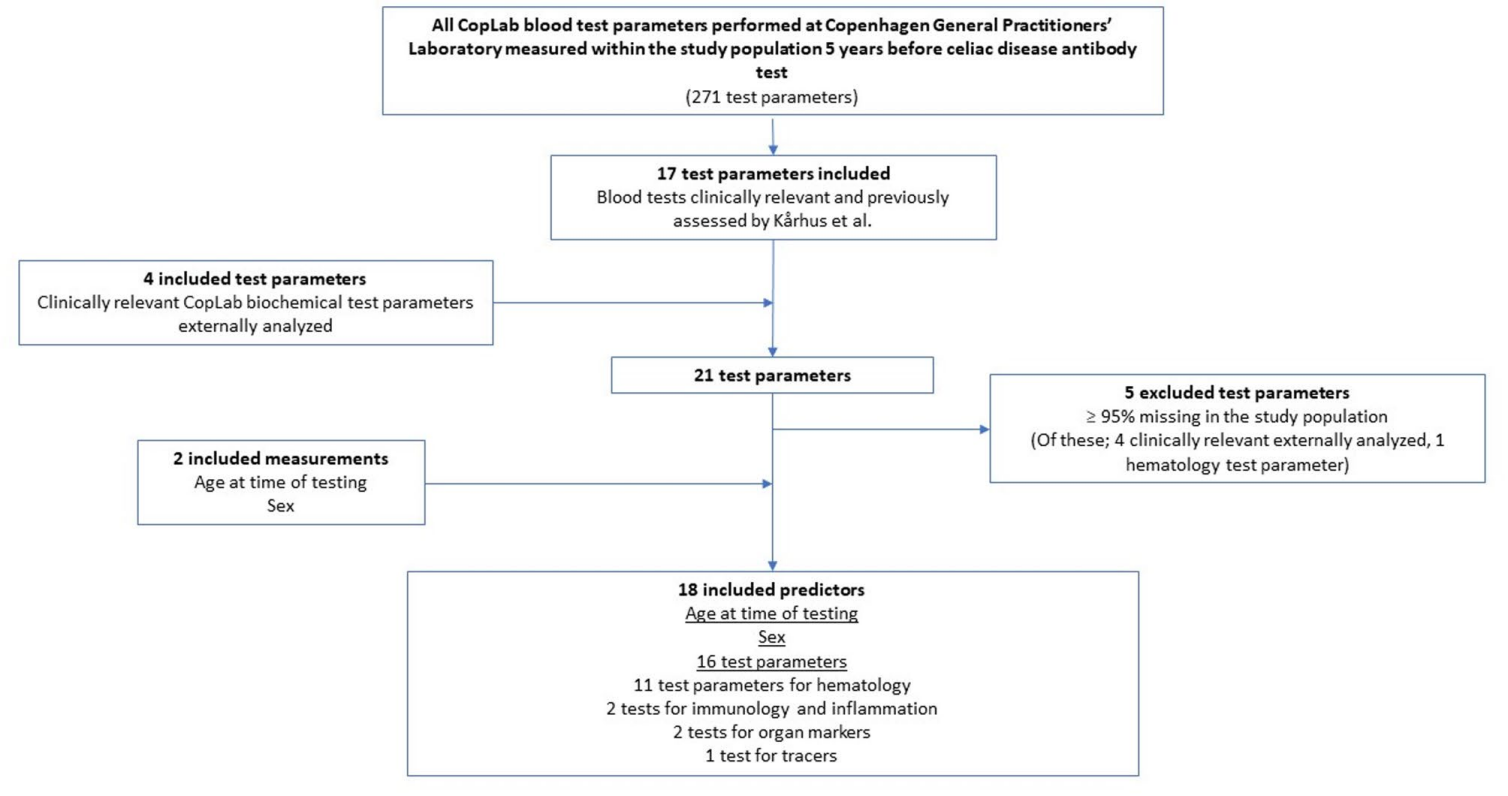
Candidate set of predictors: Curated list of test parameters.

In total, 77 predictors were included in the full list: age, sex, and 75 predictors within groups of allergy, electrolytes, endocrinology, hematology, hemostasis, immunology and inflammation, infection, metabolism, organ markers, and tracers (Fig. 2 and Supplementary Table S1). 18 predictors were included in the curated list: age, sex, and 16 different predictors within groups of hematology, immunology and inflammation, organ markers and tracers (Fig. 3 and Supplementary Table S2). As such all feature selection was concluded before model development was initiated.

### Statistical analysis

Characteristics of the subjects were summarized using proportions, means, standard deviations, and quantiles. As patients may have repeated measurement of the same test parameter during the five years of history, we summarized each biochemical test parameter by its mean, minimum, and maximum and included all of these as candidate predictors.

We used the SuperLearner approach to develop a prediction model for the probability of CD antibody positivity given the set of biochemical predictors. This involves stacking or averaging the results from a library of statistical prediction models^30^. We considered the following methods in our library of statistical models: (1) extreme gradient boosting with logistic link and log-loss and 400 boosting iterations^31^, (2) forwards stepwise variable selection using Akaike’s information criteria followed by logistic regression of the selected model^32^, (3) support vector machines with sigmoid kernel^33^, and (4) lasso logistic penalized regression including the predictors and all pairwise interactions between the candidate predictors^34^.

Most patients in our study did not have all, nor even the majority, of biochemical test parameters measured (Supplementary Table S1 and Table S2), so missing data needed to be accounted for in the model development. For the extreme gradient boosting method, which is a tree-based method, we allowed for splits on missingness. For the other methods, each missing feature was imputed by its overall mean within the cross-validation.

Each of the four models was fit using both the full list and the curated list of predictors and then we finally estimated two optimal models: (1) by stacking all eight models; both those using the curated list and those using the full list, and (2) by stacking the fitted models using only the curated list. The coefficients used to stack the models were estimated using logistic regression in a pre-validation framework^35^.

The model development process was done in a 10-fold cross-validation framework, with nested cross validation done for the LASSO method to estimate the lambda tuning parameter^36^. The cross-validation allowed us to obtain valid estimates of the receiver operating characteristic curve (ROC-curve) to assess the discrimination capacity of the models.

All analyses were conducted using R and the packages xgboost, e1071, and glmnet. Feature importance of the model was estimated using the FeatureImp function of the iml R package^37^. This fits an extreme gradient boost-based surrogate model to the outcome using the same predictors, and then randomly perturbs predictors one-by-one to see the effect on the prediction performance measured by median absolute error. We did this for the full and the curated model and summarized the 20 most important predictors according to this criterion. We also assessed the association between feature importance and the rate of missingness for each candidate predictor.

## Results

The study sample consisted of 54,877 patients; 54,205 patients were CD antibody seronegative, and 672 patients were CD antibody seropositive, yielding a prevalence of CD antibody seropositivity of 1.2%. Overall, the majority of patients were women (67.4%), also among the CD antibody seropositive (74.6%). The average age at time of CD test was 32.9 years. The average number of biochemical tests, in the five years before CD testing, was about 53, and lab testing was most frequent in the year preceding the CD test (Table 1). However, frequency of testing did not differ dramatically by CD antibody serology.

**Table 1:** Description of the study sample.

	CD antibody seropositive patients (*N* = 672)	CD antibody seronegative patients (*N* = 54,205)	Overall (*N* = 54,877)
**Sex**			
Women (%)	501 (74.6)	36,469 (67.3)	36,970 (67.4)
Men (%)	171 (25.4)	17,736 (32.7)	17,907 (32.6)
**Number of conducted biochemical tests overal**l			
Mean (SD)	47.8 (53.1)	52.7 (50.2)	52.6 (50.2)
Median [Q1, Q3]	29.0 [17.0, 59.3]	38.0 [21.0, 68.0]	38.0 [21.0, 68.0]
**Number of test parameters repeated**			
Mean (SD)	8.98 (10.4)	11.0 (10.7)	10.9 (10.7)
Median [Q1, Q3]	3.5 [0, 18.0]	11.0 [0, 19.0]	10.0 [0, 19.0]
**Number of distinct test parameters**			
Mean (SD)	23.4 (11.4)	26.0 (11.3)	26.0 (11.3)
Median [Q1, Q3]	25.0 [16.0, 31.0]	26.0 [19.0, 33.0]	26.0 [19.0, 33.0]
**Average age at CD test**			
Mean (SD)	30.1 (17.8)	32.9 (17.2)	32.9 (17.2)
Median [Q1, Q3]	26.6 [17.0, 40.4]	29.8 [21.0, 44.0]	29.7 [21.0, 43.9]
**Average number of years from first non-CD test to CD test**			
Mean (SD)	0.864 (0.997)	0.990 (0.982)	0.988 (0.982)
Median [Q1, Q3]	0.460 [0, 1.59]	0.793 [0, 1.75]	0.790 [0, 1.74]
**Median number of years from first non-CD test to CD test**			
Mean (SD)	0.792 (1.15)	0.901 (1.18)	0.900 (1.18)
Median [Q1, Q3]	0.012 [0, 1.38]	0.211 [0, 1.62]	0.208 [0, 1.62]
**Number of tests in year 0 to 1 before CD test**			
Mean (SD)	25.2 (20.2)	26.9 (19.9)	26.8 (19.9)
Median [Q1, Q3]	21.0 [13.0, 33.0]	23.0 [15.0, 35.0]	23.0 [15.0, 35.0]
**Number of tests in year 1 to 2 before CD test**			
Mean (SD)	7.0 (15.1)	7.9 (15.1)	7.9 (15.1)
Median [Q1, Q3]	0 [0, 8.0]	0 [0, 13.0]	0 [0, 13.0]
**Number of tests in year 2 to 3 before CD test**			
Mean (SD)	5.8 (13.2)	6.8 (14.1)	6.8 (14.1)
Median [Q1, Q3]	0 [0, 3.0]	0 [0, 9.0]	0 [0, 9.0]
**Number of tests in year 3 to 4 before CD test**			
Mean (SD)	5.0 (11.8)	5.9 (13.0)	5.9 (13.0)
Median [Q1, Q3]	0 [0, 1.0]	0 [0, 4.0]	0 [0, 4.0]
**Number of tests in year 4 to 5 before CD test**			
Mean (SD)	4.8 (13.2)	5.2 (12.1)	5.2 (12.2)
Median [Q1, Q3]	0 [0, 0]	0 [0, 1.0]	0 [0, 1.0]

Celiac disease * CD*, Standard deviation* SD*.

77 predictors were included in the full list, and 18 predictors were included in the curated list. Except age and sex, all predictors were summarized by three properties (minimum, mean and maximum), and therefore the full prediction model contained a total of 227 predictors, and the curated model contained 50 predictors (Supplementary Tables S1 and S2).

**Fig. 4:**
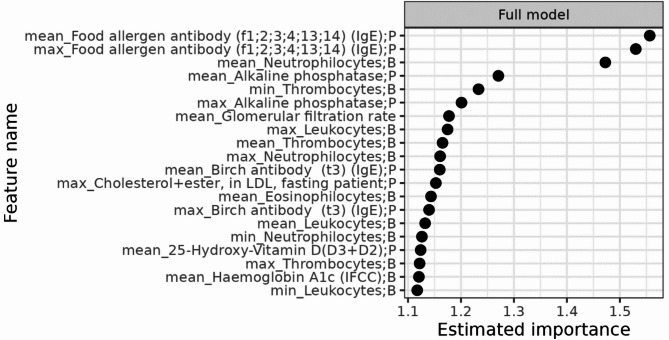
Estimated feature importance for top 20 most important test parameters predictive of a positive celiac disease antibody test in the full model.

**Fig. 5:**
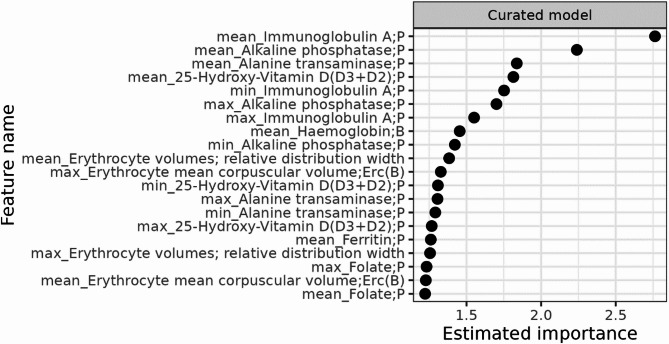
Estimated feature importance for top 20 most important test parameters predictive of a positive celiac disease antibody test in the curated model.

The estimated importance for the 20 most important variables is shown in Figs. 4 and 5 with a numeric value of importance. No single predictor had strong predictive performance, and the most important features were mean values of the food allergen antibody test parameter in the full model, and mean values of the IgA test parameter in the curated list.

**Fig. 6:**
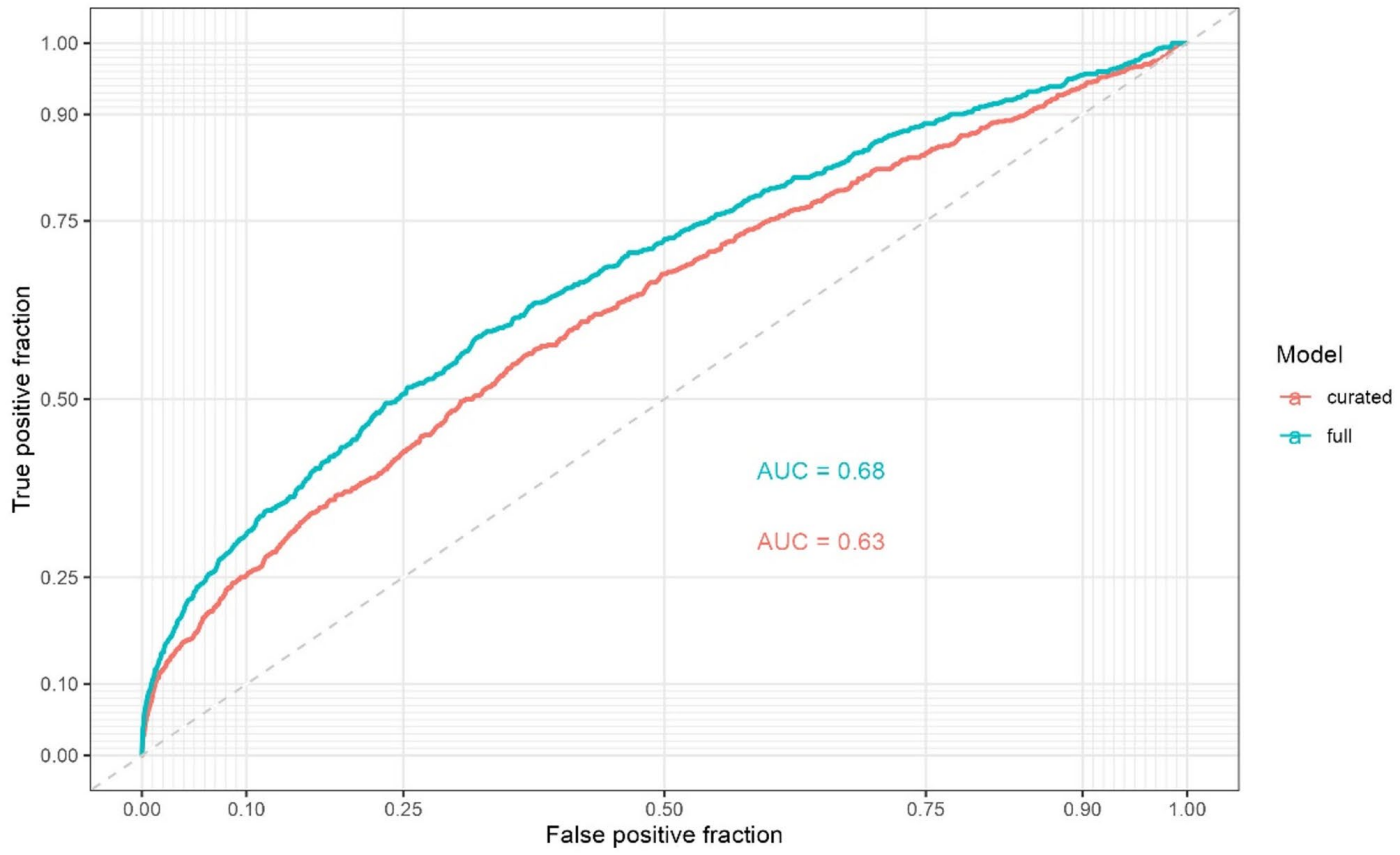
The receiver operating characteristic curves for the full and curated model. *AUC* Area under the curve.

**Fig. 7:**
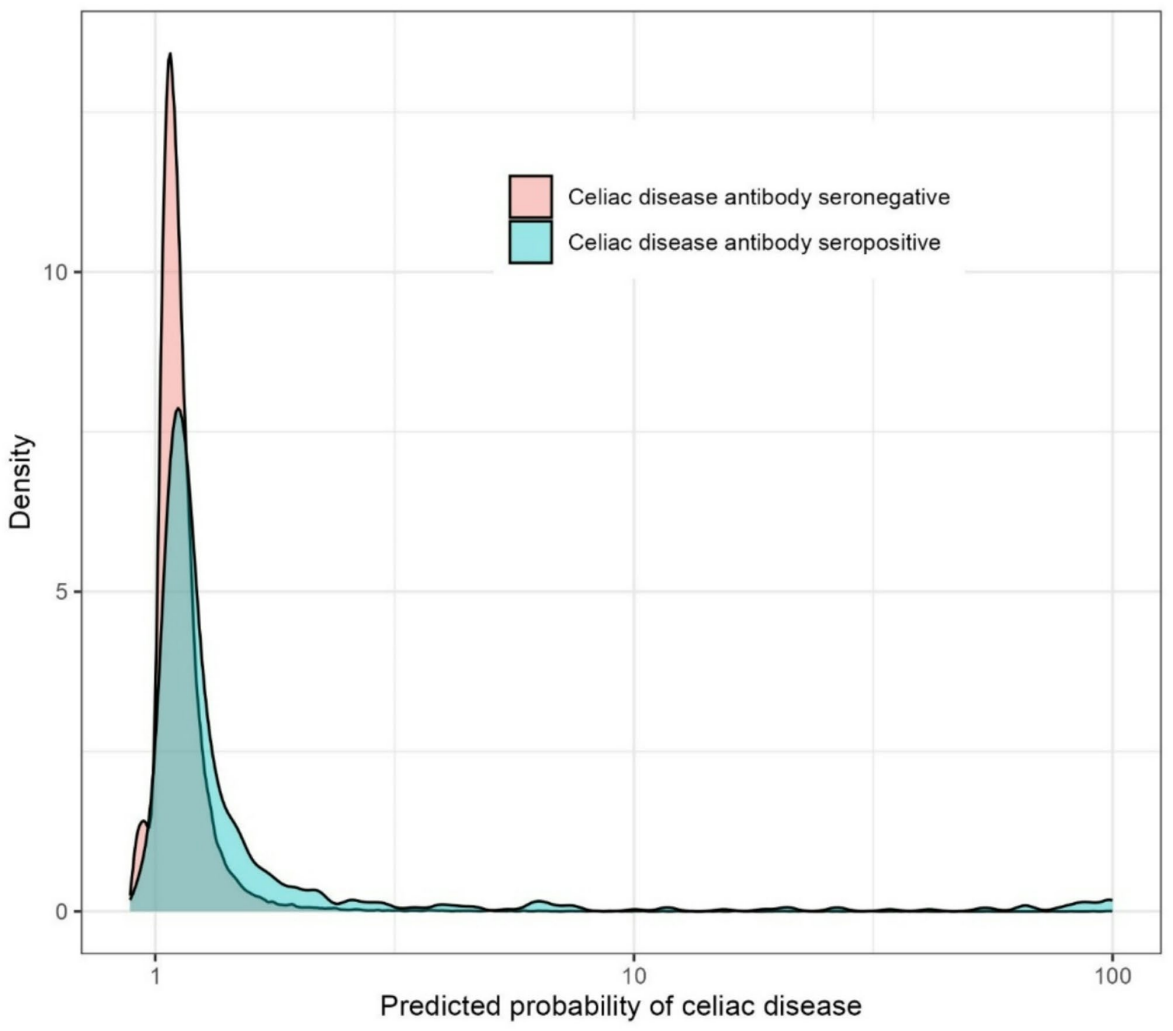
Density plots of predicted probability of celiac disease (CD) antibody positivity (log scale), by CD antibody seropositive from CD antibody seronegative patients.

Model performance was assessed by the ROC-curve methodology. The discriminative ability of the optimal stacked model, based on the full and curated model is shown in Figs. 6 and 7. The cross-validated estimated area under the curve (AUC) of the full model was 0.68 (95% confidence interval (CI): 0.66–0.70), and 0.63 (95% CI: 0.61–0.65) for the curated model. The distributions of the predicted probabilities overlapped substantially between CD antibody seropositive and seronegative patients (Fig. 7).

## Discussion

This observational cohort study included all laboratory results performed in primary care in the Copenhagen area (2006–2015) from approximately 1.3 million inhabitants as registered in the CopLab-database. The prediction models had low discriminative and predictive power in predicting CD antibody seropositivity with AUCs ranging from 0.63 to 0.68, and the most important features of the prediction models included IgA and food allergen antibody.

### Interpretation

Our models performed better than chance but would still have weak predictive and discriminative power in a clinical setting. No single predictor stood out as highly predictive. For many of the most important predictors, more statistical properties of the same test parameter were simultaneously among the most important predictors, e.g. both “min”, “mean” and “max” alkaline phosphatase in the curated model. This may indicate that the predictive power of the model, though limited, lies within the requisition of the test parameter to a larger degree than the numeric test result. The most important predictors correspond to test parameters part of the routine diagnostic process in examination of CD (mean IgA) or differential diagnostics (mean food allergen antibody). The other most important predictors, e.g. min, mean and max thrombocytes (full model) or min, mean and max 25-hydroxy-vitamin-D (curated model, mean only in full model) are examples of parameters commonly measured in primary care, and often as part of examination of more unspecific symptoms in primary care patients. Alkaline phosphatase is often measured in examination of bone diseases but also in case of suspicion of gastroenterological disease. Combined these predictors align with CD often presenting with unspecific symptoms over long time-periods, which might prompt testing for a broad variety of parameters. The mean age of the entire cohort was 32.9 years, and descriptive summarizations (Supplementary Table S1 and S2) showed all mean, minimum, and maximum values of the most important features were still within the normal ranges for adults except for food allergen antibody where the values are deviating probably due to a few high results.

We do not know why the patients encountered primary care at the time of blood tests, and therefore do not know whether the included patients had symptoms of CD. We found more frequent lab testing in the year preceding the CD test indicating a burden of disease. As the average diagnostic delay in CD is five-six years^8,9^ the inclusion of test results five years before CD antibody testing likely reflects the risk period of being symptomatic and at risk of diagnostic delay.

The association between biochemical parameters and CD antibody serology has previously been assessed in the CopLab database by Kårhus et al.^25^ with findings of biochemical numeric results differing within the normal range between CD antibody seropositive and seronegative individuals, and distinct biochemical abnormalities outside the normal range associated with CD antibody seropositivity. The parameters assessed by Kårhus et al. were the foundation of the prediction models in this study, and in line with the previous study, we found results of ferritin, hemoglobin, alanine aminotransferase, alkaline phosphatase, and mean corpuscular volume to be among the most important features of the present models. The absence of strong predictors in our models could indicate that such biochemical variations in CD may be rather unspecific, subtle, and fluctuating, as consistent patterns would most likely result in stronger predictors. As such, any variation may overlap with other diseases examined in primary care or be within the normal range and thus, also observed in healthy individuals, and may affect the models’ discriminative abilities.

In a cohort study^13^guideline-based indications to test for CD were assessed as predictors for CD in ten prediction models which had an AUC range of 0.49–0.53, thus, lower compared with the range in the present study. The authors point to the need for updated clinical guidelines, as the current clinical indications to test do not sufficiently characterize undiagnosed individuals. Other studies have assessed symptoms as predictors^14,15^. A study from primary care^15^ included symptoms and chronic conditions identified in current guidelines of CD and in a systematic review of literature as predictors^38^. The models performed well in internal validation with an AUC ranging from 0.76 to 0.82 but had low discrimination in external validation when information on first-degree relatives was absent. The authors conclude, similarly to the before mentioned cohort study^13 ^that individuals diagnosed with CD may present differently than undiagnosed, which limits prediction of CD based on guidelines, as the current guidelines are based on traits in diagnosed individuals not necessarily shared with undiagnosed CD. Such prediction models are therefore more likely to confirm predictors that are already in the guidelines and hence prompt testing but fail to identify undiagnosed individuals^15^. Similarly, as we only included patients with a CD-test in the present study, we are also likely to confirm current testing patterns and medical guidelines e.g. food allergen antibodies measured as part of CD differential diagnostics, or total IgA measured as part of CD-examination. IgA is routinely measured in CD examination in Denmark, as low IgA measures may indicate IgA-deficiency and may affect the choice of diagnostic CD-test for the clinician. We aimed to reduce the clinical selection bias by our data-driven approach in the full model including all test parameters, which showed better performance than the clinical approach in the curated model. A recent prediction model using laboratory results^24^ found high predictive power of CD seropositivity with AUC’s ranging from 0.77 to 0.86. The difference in our predictive powers may be attributed to their study predicting very high levels of CD seropositivity among clinically suspected individuals and the inclusion of controls without signs of CD. The study hereby differs substantially from the present study by study design.

### Strengths and limitations

There are several strengths to our study. We utilized a high-quality, large, and very comprehensive database with routinely collected biochemical tests from primary care performed over almost 10 years. As the diagnostic examination of CD is usually initiated in primary care, the sample of patients and predictors accurately reflect the setting and the test parameters available when deciding to examine for CD.

Furthermore, the outcome was well defined by clinically applied and valid classifications of CD with high-quality information on time for testing and prior diagnosis of CD. We included different antibody test parameters of CD making the outcome definition robust to changes in the diagnostic procedure over time. The CD prevalence in this sample did not reflect that in the general population or in secondary care, but rather the prevalence of CD among those tested for CD in primary care, and therefore we do not report metrics such as positive and negative predictive values. We conducted a decision curve analysis in post hoc analysis, as this relies on the true positives and true negatives. The results were consistent with our previous findings **(Supplementary Figure S5).** We only included patients whom the clinician had clinical suspicion of having CD. This clinical evaluation may differ among children and adults, and children might more often be examined in pediatric departments at hospitals than in primary care in case of persistent symptoms and therefore may not be well-represented in our sample. Further, we did not have information on diet, and some patients classified as antibody seronegative may be “seropositive” patients on a gluten-free diet.

Some patients were tested for CD antibodies multiple times, which may have been due to e.g. family history or comorbidities making these patients at high risk of CD. Only one test per individual was included to ensure comparable time periods for the predictors in each participant. To avoid conditioning on the future, we included the first positive test or the last negative test. The inclusion of first positive test reduced the risk of CD-measurement as part of clinical follow up in an established CD diagnosis, and the inclusion of the last negative test ensured assessment of all results over the period with clinical suspicion of incident CD.

Due to the large number of predictors available and to reduce clinical selection bias, we included two sets of candidate predictors testing two different approaches, which is another strength to the study. The more objective and data-driven approach in the full model performed better than the more clinical-driven approach indicating that the model found nuances that clinicians may not. However, as the difference in performance was small, the potential clinical selection bias was likely limited.

As all patients tested for CD in primary care have individual examinations and risk assessments depending on the clinician’s choices in the specific case, all patients will have different biochemical test parameters measured at different time points. This results in large amounts of missing data, which was a limitation in our study. Test parameters with proportions missing in 95% or more of the study population were excluded, but proportions missing still reached about 94% in some test parameters (Supplementary Tables S1 and S2). However, in post hoc analyses, we did not find a correlation between proportions missing and predictive importance (Supplementary Figures S3 and S4). We accounted for missingness by imputation of the overall mean without considering the CD antibody serology. Imputing the mean for such a large amount of missing data results in reduced variance within the dataset, which might have decreased the variability and thereby the discriminating power of the models. However, if we had applied the mean of the parameters within groups of CD antibody serology status the model would not have been clinically implementable, as the clinician at time of the biochemical test would not know the CD antibody serology status. When applying the median, instead of the mean, in post hoc analyses, the AUCs decreased slightly to AUCs 0.65 (full) and 0.64 (curated). In post hoc analyses, we also found no alteration of the AUCs when assessing shorter time spans prior to CD-test (Supplementary Figure S6). When assessing the AUCs for the individual models instead for stacked, the AUCs ranged from 0.55 to 0.64 for the curated model and 0.55–0.68 for the full model, with no one model standing out (Supplementary Table S7). As such, as we used a broad range of statistical approaches and variable selections, we would be able to identify a well-performing model if one were possible to identify in the data. Furthermore, SHAP analyses yielded similar test parameters as the feature importance model (Supplementary Table S8).

We summarized the parameters by the measured mean, minimum, and maximum to keep the essence of the dynamic properties of the parameters in the models (Supplementary Tables S1 and S2). We did not categorize by abnormalities or reference-intervals, as such categorization would result in loss of the nuances within the numeric values. Due to the large number of predictors, we needed to account for the risk of overfitting. The risk was minimized through the statistical methods including extreme gradient boosting, stepwise variable selection, and lasso logistic penalized regression.

The diagnostic process is highly complex and variable, and the models lack information on the clinical evaluation and background for testing made by the clinician. Whether the clinician’s decision to test was based on biochemical results, clinical findings, subjective symptoms, other chronic conditions, or family history remains unknown. The comparison group, being CD antibody seronegative patients encountering primary care, may have been examined for other diseases that could explain their testing patterns and test results. As such, important predictive and discriminative information may be lost when only assessing biochemical test parameters alone, which is a limitation. However, as previous models based on subjective measurements of symptoms and risk factors have had poorer prediction, we aimed to explore whether objective measurements would provide a more feasible approach, and therefore we kept the predictors restricted to objective information at hand of the general practitioner. If it were possible to combine subjective measurements in combination with objective measurements of biochemical or genetics tests, such model taking e.g. the entire diagnostic process into account, would be at risk of becoming a highly specialized and overfit prediction model lacking generalizability and potential for clinical implementation.

### Perspectives

Long diagnostic delays in CD pose a significant clinical problem and prediction models may play a role in reducing it. However, such models must consider that biochemical results in CD may be subtle, fluctuating, and unspecific why biochemical results, when on their own, appear weak numeric predictors of CD. Further, the risk of clinical selection bias makes it highly relevant to carefully consider the predictors, setting and study population assessed.

We explored biochemical test parameters as objective predictors to decrease the diagnostic delay in CD, and despite the poor performance, we believe our study contributes with valuable methodological insights for future studies. Future models may benefit from combing biochemical results with family history, comorbidities, symptoms, frequency of consultations and/or genetic traits. Such models will have to balance higher predictive power in relation to generalizability, as they are at risk of becoming less clinically implementable. However, as the diagnostic delay is currently long, insights from these models may still play important parts in better risk identification.

## Conclusion

In this observational cohort study, we developed two prediction models to predict CD antibody seropositivity, based on routinely collected biochemical tests in primary care. The biochemical tests were weak predictors on their own, but the models provide novel insights into the methodologies of utilizing objective test parameters as predictors of CD. Future prediction models may benefit from combining biochemical tests and other clinical information but must balance between having higher predictive power and being clinically implementable.

## Electronic supplementary material

Below is the link to the electronic supplementary material.

Supplementary Material 1

## Data Availability

Restrictions apply to the availability of these data according to Danish legislation. Therefore, the data and the information regarding the patients cannot be publicly available. Access to these data needs approval from the Danish authorities. Data are stored at a server at University of Copenhagen, and information on data access from the CopLab cohort is provided by the steering group upon request (www.publichealth.ku.dk/research/databases-for-collaboration/coplab/).
