# Supplementary material for: Machine learning-based prediction of celiac antibody seropositivity by biochemical test parameters
Source: Sci Rep. 2025 Jul 3;15:23732. doi: 10.1038/s41598-025-08225-6 (PMC12229532; doi:10.1038/s41598-025-08225-6)
Supplement: Supplementary file 1 — Supplementary Material 1 [file 41598_2025_8225_MOESM1_ESM.pdf]

Machine learning-based prediction of celiac antibody seropositivity by biochemical test parameters

Signe Ulfbeck Schovsbo*[1], Michael Charles Sachs[2], Margit Kriegbaum[3], Anne Ahrendt Bjerregaard[1], Line Tang Møllehave[1], Susanne Hansen[1], Bent Struer Lind[4], Tora Grauers Willadsen[3], Allan Linneberg[1,5], Christen Lykkegaard Andersen[3,6], Line Lund Kårhus[1]

List of affiliations

[1] Center for Clinical Research and Prevention, Copenhagen University Hospital – Bispebjerg and Frederiksberg, Copenhagen, Denmark.

[2] Section of Biostatistics, Department of Public Health, University of Copenhagen, Copenhagen, Denmark.

[3] Copenhagen Primary Care Laboratory (CopLab) Database, Research Unit for General Practice and Section of General Practice, Department of Public Health, University of Copenhagen, Copenhagen, Denmark.

[4] Department of Clinical Biochemistry, Copenhagen University Hospital Hvidovre, Hvidovre, Denmark.

[5] Department of Clinical Medicine, Faculty of Health and Medical Science, University of Copenhagen, Copenhagen C, Denmark.

[6] Department of Hematology, Rigshospitalet, Copenhagen, Denmark

Corresponding author

*Signe Ulfbeck Schovsbo

Center for Clinical Research and Prevention, Copenhagen University Hospital – Bispebjerg and Frederiksberg, Copenhagen, Denmark.

E-mail: signe.ulfbeck.schovsbo@regionh.dk

Word count

Abstract: 199 words (max 200 words)

Full text: 4.578 (max 4500 words)

Supplementary table S1: Overview of the of candidate predictors in the full list

Analysis group	English name	International Union of Pure and Applied Chemistry	Statistic property	Mean	Standard deviation	Q1	Median	Q3	Proportion missing	Note
Immunology and inflammation	Immunoglobulin A;P	NPU19795	mean	2.096	0.929	1.45	1.97	2.60	0.011	
			min	2.077	0.925	1.44	1.95	2.57	0.011	
			max	2.115	0.938	1.46	1.98	2.62	0.011	
Hematology	Erythrocyte mean corpuscular volume;Erc(B)	NPU01944	mean	88.783	5.419	86.00	89.00	92.00	0.084	
			min	87.617	5.526	85.00	88.00	91.00	0.084	
			max	89.996	5.812	87.00	90.00	94.00	0.084	
Hematology	Leukocytes;B	NPU02593	mean	6.988	2.050	5.70	6.70	8.00	0.084	
			min	6.291	1.815	5.00	6.00	7.20	0.084	
			max	7.813	2.930	6.10	7.40	9.00	0.084	
Hematology	Erythrocyte volumes; relative distribution width	NPU18162	mean	13.157	0.877	12.68	13.05	13.48	0.084	
			min	12.887	0.738	12.40	12.80	13.20	0.084	
			max	13.460	1.761	12.80	13.30	13.80	0.084	
Hematology	Haemoglobin;B	NPU02319	mean	8.401	0.741	7.90	8.33	8.88	0.084	
			min	8.201	0.816	7.70	8.20	8.70	0.084	
			max	8.597	0.754	8.10	8.50	9.10	0.084	
Hematology	Thrombocytes;B	NPU03568	mean	281.530	66.730	236.00	274.00	319.00	0.084	
			min	264.753	65.199	221.00	257.00	301.00	0.084	
			max	300.506	78.372	248.00	290.00	341.00	0.084	
Endocrinology	Thyrotropin;P	NPU03577	mean	1.973	2.923	1.20	1.60	2.28	0.147	
			min	1.622	2.446	0.96	1.40	2.00	0.147	
			max	2.503	5.449	1.30	1.80	2.60	0.147	
Organ markers	Alanine transaminase;P	NPU19651	mean	25.347	19.522	16.67	21.00	28.00	0.168	

			min	21.404	15.420	15.00	18.00	24.00	0.168
			max	31.070	37.999	18.00	23.00	33.00	0.168
Immunology and inflammation	C-reactive protein;P	NPU19748	mean	6.500	8.648	4.00	4.00	5.00	0.190
			min	5.204	6.677	4.00	4.00	5.00	0.190
			max	8.920	16.729	4.00	4.00	5.00	0.190
Organ markers	Alkaline phosphatase;P	NPU19655	mean	38.019	29.394	22.73	28.33	36.67	0.214
			min	35.791	28.700	21.25	26.25	34.17	0.214
			max	40.582	32.085	23.75	30.00	40.00	0.214
Hematology	Eosinophilocytes; B	NPU01933	mean	0.214	0.198	0.11	0.17	0.26	0.259
			min	0.184	0.183	0.09	0.14	0.22	0.259
			max	0.248	0.251	0.12	0.19	0.30	0.259
Hematology	Lymphocytes;B	NPU02636	mean	2.179	1.207	1.69	2.06	2.52	0.259
			min	2.017	1.047	1.54	1.91	2.37	0.259
			max	2.347	1.439	1.79	2.20	2.71	0.259
Hematology	Monocytes;B	NPU02840	mean	0.401	0.141	0.31	0.38	0.47	0.259
			min	0.367	0.133	0.28	0.35	0.43	0.259
			max	0.440	0.180	0.33	0.41	0.51	0.259
Hematology	Neutrophilocytes ;B	NPU02902	mean	3.982	1.628	2.90	3.69	4.71	0.259
			min	3.559	1.539	2.55	3.27	4.21	0.259
			max	4.488	2.135	3.08	4.04	5.35	0.259
Hematology	Basophilocytes;B	NPU01349	mean	0.037	0.018	0.02	0.03	0.05	0.259
			min	0.033	0.017	0.02	0.03	0.04	0.259
			max	0.043	0.025	0.03	0.04	0.05	0.259
Electrolytes	Sodium ion;P	NPU03429	mean	140.739	1.746	140.00	141.00	142.00	0.273
			min	140.045	2.092	139.00	140.00	141.00	0.273
			max	141.445	2.001	140.00	141.00	143.00	0.273
Electrolytes	Potassium ion;S	NPU03230	mean	4.291	0.267	4.10	4.30	4.45	0.279
			min	4.187	0.295	4.00	4.20	4.40	0.279
			max	4.399	0.326	4.20	4.40	4.60	0.279

Organ markers	Glomerular filtration; vol.rate(ml/min per surf.=1.73 m²);Kidn.	DNK35131	mean	104.668	17.079	93.58	106.00	117.67	0.314
			min	100.938	19.001	89.00	103.00	115.00	0.314
			max	108.086	16.317	97.00	109.00	121.00	0.314
Tracers and vitamins	25-Hydroxy-Vitamin D(D3+D2);P	NPU10267	mean	61.930	25.106	45.00	59.80	76.27	0.393
			min	53.324	26.868	33.00	50.60	69.30	0.393
			max	71.064	30.834	51.00	68.20	88.00	0.393
Endocrinology	Calcium;P	NPU01443	mean	2.338	0.088	2.28	2.34	2.39	0.479
			min	2.315	0.095	2.25	2.31	2.38	0.479
			max	2.362	0.096	2.30	2.36	2.42	0.479
Organ markers	Albumin;P	NPU19673	mean	46.673	2.433	45.04	46.68	48.23	0.534
			min	46.338	2.598	44.74	46.49	47.84	0.534
			max	47.008	2.482	45.52	46.87	48.62	0.534
Hematology	Cobalamin;P	NPU01700	mean	318.458	136.044	230.00	290.00	370.00	0.552
			min	301.295	130.338	220.00	275.00	355.00	0.552
			max	338.881	166.864	240.00	300.00	390.00	0.552
Organ markers	Amylase;P	NPU19652	mean	18.466	7.283	13.94	17.42	21.82	0.560
			min	17.961	7.043	13.33	16.97	21.21	0.560
			max	19.032	8.031	14.24	17.88	22.42	0.560
Organ markers	Bilirubins;P	NPU01370	mean	10.279	5.814	7.00	9.00	12.00	0.579
			min	9.661	5.555	6.00	8.00	11.00	0.579
			max	10.936	6.642	7.00	9.00	13.00	0.579
Metabolism	Cholesterol+ester;P	NPU01566	mean	4.990	0.968	4.30	4.90	5.60	0.587
			min	4.745	0.941	4.10	4.70	5.30	0.587
			max	5.246	1.144	4.40	5.10	6.00	0.587
Organ markers	gamma-Glutamyltransferase;P	NPU19657	mean	29.660	54.547	12.77	18.09	28.72	0.595

			min	25.933	44.046	11.70	17.02	26.60	0.595
			max	34.498	82.349	12.77	19.15	30.85	0.595
Metabolism	Haemoglobin A1c (IFCC);B	NPU27300	mean	35.000	6.336	31.50	34.00	37.00	0.629
			min	34.250	5.488	31.00	34.00	37.00	0.629
			max	35.845	8.127	32.00	34.00	38.00	0.629
Metabolism	Cholesterol+ester. in HDL;P	NPU01567	mean	1.500	0.423	1.19	1.44	1.75	0.636
			min	1.429	0.416	1.12	1.37	1.67	0.636
			max	1.572	0.458	1.24	1.51	1.83	0.636
Hematology	Haemoglobin. MCHC;Erc(B)	NPU02321	mean	20.739	0.679	20.35	20.80	21.20	0.651
			min	20.608	0.764	20.20	20.70	21.10	0.651
			max	20.868	0.686	20.50	20.90	21.30	0.651
Hematology	Ferritin;P	NPU19763	mean	78.175	103.140	25.00	46.00	95.00	0.672
			min	73.048	98.388	22.00	42.00	90.00	0.672
			max	84.151	115.604	27.00	50.50	102.00	0.672
Metabolism	Triglyceride. fasting patient;P	NPU03620	mean	1.316	0.922	0.79	1.10	1.58	0.727
			min	1.152	0.809	0.71	0.97	1.38	0.727
			max	1.518	1.285	0.84	1.21	1.80	0.727
Hematology	Iron;P	NPU02508	mean	16.167	6.727	12.00	16.00	20.00	0.785
			min	15.484	6.953	11.00	15.00	19.00	0.785
			max	16.916	7.195	12.00	16.00	21.00	0.785
Metabolism	Glucose. patient fasting status unknown;P	NPU02192	mean	5.525	1.215	4.95	5.30	5.80	0.787
			min	5.424	1.175	4.90	5.20	5.70	0.787
			max	5.633	1.361	5.00	5.40	5.90	0.787
Metabolism	Glucose. patient fasting status unknown;S	KPL00291	mean	5.462	1.110	4.90	5.30	5.80	0.796
			min	5.325	1.076	4.80	5.20	5.60	0.796
			max	5.610	1.277	5.00	5.40	5.90	0.796

Haemostasis	Coagulation. tissue factor-induced;P	NPU18878	mean	0.976	0.195	0.85	0.96	1.10	0.816
			min	0.970	0.196	0.84	0.96	1.09	0.816
			max	0.983	0.197	0.85	0.97	1.10	0.816
Hematology	Transferrin;P	NPU03607	mean	33.728	5.832	30.00	33.00	37.00	0.820
			min	33.378	5.809	29.00	33.00	36.00	0.820
			max	34.089	6.070	30.00	33.00	37.00	0.820
Allergy	Food allergen antibody (f1;2;3;4;13;14)(IgE);P	NPU19617	mean	0.343	3.001	0.04	0.08	0.34	0.830
			min	0.319	2.877	0.03	0.07	0.34	0.830
			max	0.372	3.366	0.04	0.08	0.34	0.830
Metabolism	Triglyceride. patient fasting status unknown;P	NPU04094	mean	1.416	0.920	0.84	1.17	1.70	0.830
			min	1.301	0.845	0.77	1.07	1.56	0.830
			max	1.551	1.146	0.87	1.25	1.87	0.830
Metabolism	Cholesterol+ester. in LDL. fasting patient;P	NPU10171	mean	2.984	0.851	2.38	2.94	3.53	0.838
			min	2.836	0.853	2.23	2.79	3.38	0.838
			max	3.143	0.949	2.47	3.06	3.74	0.838
Hematology	Transferrin(Iron binding sites;Plasma)—Iron; substance fraction = ?	NPU04191	mean	0.250	0.115	0.17	0.24	0.31	0.839
			min	0.242	0.117	0.16	0.23	0.31	0.839
			max	0.259	0.119	0.18	0.25	0.33	0.839
Immunology and inflammation	Immunoglobulin G;P	NPU19814	mean	10.185	2.295	8.60	10.00	11.50	0.851
			min	10.102	2.288	8.60	9.90	11.40	0.851
			max	10.268	2.337	8.70	10.10	11.60	0.851

Immunology and inflammation	Immunoglobulin M;P	NPU19825	mean	1.028	0.572	0.69	0.93	1.24	0.851
			min	1.014	0.564	0.68	0.91	1.23	0.851
			max	1.042	0.583	0.70	0.94	1.26	0.851
Allergy	Inhalation antigen antibody;P	NPU27315	mean	7.155	18.120	0.04	0.11	3.07	0.851
			min	7.023	17.908	0.04	0.11	2.95	0.851
			max	7.291	18.417	0.04	0.12	3.11	0.851
Endocrinology	Parathyroid hormone;P	NPU03028	mean	5.184	2.759	3.40	4.70	6.30	0.853
			min	4.837	2.590	3.10	4.40	5.90	0.853
			max	5.566	3.356	3.50	4.90	6.80	0.853
Immunology and inflammation	Erytrocyte sedimentation reaction;B	NPU17589	mean	9.391	9.306	4.00	7.00	12.00	0.857
			min	8.633	8.628	3.00	6.00	11.00	0.857
			max	10.305	11.062	4.00	7.00	13.00	0.857
Endocrinology	Thyroxine. free;P	NPU03579	mean	15.319	3.698	13.60	14.95	16.40	0.874
			min	14.411	3.622	12.80	14.30	15.80	0.874
			max	16.364	5.355	13.90	15.40	17.30	0.874
Metabolism	Urate;P	NPU03688	mean	0.299	0.083	0.24	0.29	0.35	0.877
			min	0.291	0.081	0.23	0.28	0.34	0.877
			max	0.307	0.089	0.24	0.29	0.36	0.877
Metabolism	Glucose. fasting patient;P	NPU02195	mean	5.757	1.356	5.10	5.45	5.90	0.882
			min	5.611	1.198	5.00	5.40	5.80	0.882
			max	5.919	1.686	5.10	5.50	6.00	0.882
Metabolism	Glucose. fasting patient;S	KPL00290	mean	5.544	1.145	5.00	5.32	5.80	0.890
			min	5.421	1.047	4.90	5.30	5.70	0.890
			max	5.679	1.376	5.00	5.40	5.90	0.890
Immunology and inflammation	Rheumatoid factor;P	NPU18350	mean	12.517	11.121	10.00	10.00	11.00	0.894
			min	12.219	10.559	10.00	10.00	10.00	0.894

			max	12.845	11.921	10.00	10.00	11.00	0.894
Infection	Epstein-Barr virus nucelar antibody (IgG);P	NPU12471	mean	169.989	220.830	2.00	38.00	308.00	0.895
			min	167.370	221.126	2.00	29.00	305.00	0.895
			max	172.606	222.875	2.00	39.00	318.00	0.895
Immunology and inflammation	Orosomucoid;P	NPU19873	mean	0.836	0.282	0.65	0.79	0.97	0.903
			min	0.830	0.282	0.64	0.79	0.97	0.903
			max	0.842	0.288	0.65	0.80	0.98	0.903
Endocrinology	Follitropin;P	NPU04014	mean	16.074	24.246	4.10	6.20	11.80	0.905
			min	14.255	23.207	3.40	5.60	9.50	0.905
			max	18.006	26.970	4.30	6.70	13.60	0.905
Allergy	Dermatophagoides pteronyssinus antibody (d1) (IgE);P	NPU10881	mean	7.063	17.987	0.06	0.34	3.45	0.915
			min	6.877	17.766	0.06	0.34	3.17	0.915
			max	7.253	18.326	0.07	0.34	3.53	0.915
Allergy	Birch antibody (t3) (IgE);P	NPU10842	mean	6.703	18.242	0.02	0.34	2.33	0.916
			min	6.418	17.782	0.02	0.34	2.16	0.916
			max	7.017	19.077	0.02	0.34	2.41	0.916
Allergy	Timothy grass antibody (g6) (IgE);P	NPU10899	mean	9.486	21.548	0.07	0.41	6.07	0.916
			min	9.174	21.199	0.06	0.38	5.83	0.916
			max	9.810	22.121	0.07	0.41	6.22	0.916
Allergy	Cat dander antibody (e1) (IgE);P	NPU11028	mean	1.857	8.074	0.01	0.12	0.37	0.918
			min	1.784	7.886	0.01	0.10	0.34	0.918
			max	1.933	8.411	0.01	0.12	0.37	0.918

Allergy	Mugwort antibody (w6) (IgE);P	NPU10936	mean	0.990	4.904	0.02	0.13	0.34	0.919
			min	0.944	4.742	0.02	0.11	0.34	0.919
			max	1.035	5.135	0.02	0.14	0.34	0.919
Allergy	Dog dander antibody (e5) (IgE);P	NPU10968	mean	0.875	4.577	0.04	0.19	0.34	0.919
			min	0.833	4.476	0.04	0.17	0.34	0.919
			max	0.917	4.755	0.04	0.19	0.34	0.919
Allergy	Horse dander antibody (e3) (IgE);P	NPU10958	mean	0.551	3.732	0.02	0.06	0.34	0.921
			min	0.527	3.648	0.02	0.05	0.34	0.921
			max	0.575	3.859	0.02	0.06	0.34	0.921
Endocrinology	Lutropin;P	NPU02618	mean	10.656	13.083	3.20	5.73	11.90	0.921
			min	9.483	12.636	2.60	5.00	9.80	0.921
			max	12.004	14.827	3.50	6.30	13.70	0.921
Hematology	Folate;P	NPU02070	mean	20.457	11.943	11.96	17.20	25.70	0.923
			min	19.614	11.919	11.30	16.40	24.60	0.923
			max	21.363	12.756	12.20	17.80	26.78	0.923
Allergy	Mold fungus antibody (m1;2;3;6) (IgE);P	NPU19626	mean	0.706	3.778	0.05	0.08	0.27	0.927
			min	0.671	3.525	0.05	0.08	0.25	0.927
			max	0.742	4.133	0.05	0.08	0.34	0.927
Endocrinology	Estradiol;P	NPU01972	mean	0.302	0.483	0.08	0.18	0.38	0.929
			min	0.261	0.462	0.07	0.14	0.31	0.929
			max	0.354	0.598	0.09	0.20	0.43	0.929
Infection	Hepatitis B virus s antibody;P	NPU16065	mean	114.599	277.465	7.40	7.40	7.40	0.932
			min	110.889	275.479	7.40	7.40	7.40	0.932
			max	118.264	283.820	7.40	7.40	7.40	0.932
Infection	Hepatitis B virus s antigen;P	NPU02349	mean	0.008	0.089	0.00	0.00	0.00	0.933

			min	0.008	0.089	0.00	0.00	0.00	0.933
			max	0.008	0.089	0.00	0.00	0.00	0.933
Hematology	Reticulocytes;B	NPU08694	mean	62.018	22.558	47.40	58.88	72.51	0.937
			min	59.758	22.470	45.00	56.60	70.20	0.937
			max	64.435	24.550	48.68	60.60	75.63	0.937
Hematology	Reticulcyte. haemoglobin;Rtcs(B)	NPU17007	mean	1.871	0.201	1.78	1.92	2.00	0.937
			min	1.855	0.215	1.76	1.91	2.00	0.937
			max	1.887	0.197	1.81	1.93	2.01	0.937
Immunology and inflammation	Nucleus antibody (IgG);P	NPU14127	mean	0.492	2.500	0.10	0.20	0.30	0.937
			min	0.479	2.470	0.10	0.20	0.30	0.937
			max	0.506	2.533	0.10	0.20	0.30	0.937
Immunology and inflammation	DNA(double stranded) antibody (IgG);P	NPU16393	mean	2.383	7.746	0.60	1.00	2.00	0.938
			min	2.302	7.165	0.60	0.90	1.90	0.938
			max	2.469	8.530	0.60	1.00	2.00	0.938
Endocrinology	Prolactin;P	NPU18247	mean	220.722	193.580	130.00	180.00	255.00	0.939
			min	206.850	182.472	125.00	170.00	240.00	0.939
			max	236.153	222.164	135.00	185.00	265.00	0.939
Metabolism	Glucose. fasting patient;P	DNK35842	mean	5.663	1.185	5.10	5.40	5.90	0.939
			min	5.585	1.106	5.10	5.40	5.80	0.939
			max	5.744	1.350	5.10	5.50	6.00	0.939
Endocrinology	Choriogonadotropin+beta-chain;P	NPU19579	mean	2915.764	13018.946	2.00	2.00	10.00	0.945
			min	1693.990	11542.848	2.00	2.00	10.00	0.945
			max	4692.378	18023.337	2.00	2.00	10.00	0.945
Haemostasis	Coagulation. tissue factor-induced (INR);P	NPU01685	mean	1.099	0.432	0.90	1.00	1.10	0.947
			min	1.040	0.295	0.90	1.00	1.00	0.947

			max	1.205	0.902	0.90	1.00	1.10	0.947	
Endocrinology	Progesterone;P	NPU03242	mean	12.012	23.472	1.00	3.00	15.00	0.948	
			min	9.651	18.117	1.00	2.00	7.00	0.948	
			max	14.959	43.095	1.00	3.00	19.00	0.948	
Allergy	Nut antibody (f13;17;18;20;36) (IgE);P	NPU26712	mean	2.817	11.160	0.09	0.14	0.34	0.950	Excluded because of proportion missing ≥ 95%
			min	2.731	10.968	0.09	0.14	0.34	0.950	Excluded because of proportion missing ≥ 95%
			max	2.921	11.610	0.09	0.14	0.34	0.950	Excluded because of proportion missing ≥ 95%
Endocrinology	Triiodothyronine. total;P	NPU03624	mean	1.852	0.560	1.50	1.80	2.10	0.950	Excluded because of proportion missing ≥ 95%
			min	1.778	0.565	1.40	1.70	2.10	0.950	Excluded because of proportion missing ≥ 95%
			max	1.941	0.646	1.58	1.80	2.20	0.950	Excluded because of proportion missing ≥ 95%
Infection	Epstein-Barr virus capsid antibody (IgM);P	NPU14079	mean	11.566	32.663	0.00	0.00	10.00	0.950	Excluded because of proportion missing ≥ 95%
			min	11.037	32.128	0.00	0.00	10.00	0.950	Excluded because of proportion missing ≥ 95%
			max	12.124	34.204	0.00	0.00	10.00	0.950	Excluded because of

										proportion missing ≥ 95%
Tumor marker	Prostata specific antigen;P	NPU08669	mean	1.472	2.900	0.50	0.80	1.40	0.951	Excluded because of proportion missing ≥ 95%
			min	1.258	2.530	0.50	0.80	1.30	0.951	Excluded because of proportion missing ≥ 95%
			max	1.754	3.746	0.50	0.90	1.60	0.951	Excluded because of proportion missing ≥ 95%
Allergy	Cow's milk antibody (f2) (IgE);P	NPU11037	mean	0.270	0.884	0.02	0.06	0.34	0.953	Excluded because of proportion missing ≥ 95%
			min	0.263	0.873	0.02	0.06	0.34	0.953	Excluded because of proportion missing ≥ 95%
			max	0.281	0.928	0.02	0.06	0.34	0.953	Excluded because of proportion missing ≥ 95%
Allergy	Gluten antibody (f79) (IgE);P	NPU10926	mean	0.135	0.600	0.01	0.03	0.17	0.954	Excluded because of proportion missing ≥ 95%
			min	0.131	0.554	0.01	0.03	0.15	0.954	Excluded because of proportion missing ≥ 95%
			max	0.139	0.667	0.01	0.03	0.17	0.954	Excluded because of proportion missing ≥ 95%

Endocrinology	Iodide peroxidase antibody;P	NPU20041	mean	432.025	551.021	33.00	53.00	1300.00	0.955	Excluded because of proportion missing ≥ 95%
			min	418.476	547.250	30.00	52.00	1300.00	0.955	Excluded because of proportion missing ≥ 95%
			max	444.794	560.477	33.00	56.00	1300.00	0.955	Excluded because of proportion missing ≥ 95%
Allergy	Food allergen antibody (f3;24;37;40;41)(IgE);P	NPU26713	mean	0.333	2.340	0.01	0.03	0.20	0.957	Excluded because of proportion missing ≥ 95%
			min	0.325	2.274	0.01	0.03	0.20	0.957	Excluded because of proportion missing ≥ 95%
			max	0.342	2.432	0.01	0.03	0.24	0.957	Excluded because of proportion missing ≥ 95%
Organ markers	Lactate dehydrogenase;P	NPU19658	mean	87.639	23.303	73.33	83.45	98.57	0.957	Excluded because of proportion missing ≥ 95%
			min	86.964	23.276	72.86	82.86	97.62	0.957	Excluded because of proportion missing ≥ 95%
			max	88.369	23.677	73.81	83.81	99.52	0.957	Excluded because of proportion missing ≥ 95%
Allergy	Immunoglobulin E;P	NPU02482	mean	152.567	401.400	11.00	32.00	115.00	0.958	Excluded because of

										proportion missing ≥ 95%
			min	147.286	371.502	11.00	32.00	112.25	0.958	Excluded because of proportion missing ≥ 95%
			max	158.063	465.827	11.00	32.00	117.00	0.958	Excluded because of proportion missing ≥ 95%
Infection	Human hepatitis A virus antibody (IgM);P	NPU12028	mean	0.778	0.202	0.79	0.79	0.79	0.962	Excluded because of proportion missing ≥ 95%
			min	0.776	0.207	0.79	0.79	0.79	0.962	Excluded because of proportion missing ≥ 95%
			max	0.779	0.201	0.79	0.79	0.79	0.962	Excluded because of proportion missing ≥ 95%
Infection	Human hepatitis A virus antibody;P	NPU16102	mean	58.630	39.523	20.00	46.00	100.00	0.962	Excluded because of proportion missing ≥ 95%
			min	58.246	39.735	20.00	36.00	100.00	0.962	Excluded because of proportion missing ≥ 95%
			max	58.981	39.664	20.00	46.50	100.00	0.962	Excluded because of proportion missing ≥ 95%
Endocrinology	Thyroxine. total;P	NPU03578	mean	108.977	24.668	92.00	107.00	122.00	0.965	Excluded because of proportion missing ≥ 95%

			min	105.612	25.303	90.00	104.00	120.00	0.965	Excluded because of proportion missing ≥ 95%
			max	112.564	28.322	94.00	108.00	127.00	0.965	Excluded because of proportion missing ≥ 95%
Infection	Rubellavirus antibody (IgG);P	NPU12410	mean	200.774	170.324	66.00	139.00	311.00	0.969	Excluded because of proportion missing ≥ 95%
			min	198.269	170.331	63.00	135.00	301.00	0.969	Excluded because of proportion missing ≥ 95%
			max	203.458	171.425	66.00	140.00	318.00	0.969	Excluded because of proportion missing ≥ 95%
Allergy	Wheat antibody (f4) (IgE);P	NPU10978	mean	0.830	3.755	0.04	0.12	0.35	0.970	Excluded because of proportion missing ≥ 95%
			min	0.777	3.583	0.04	0.11	0.34	0.970	Excluded because of proportion missing ≥ 95%
			max	0.901	4.107	0.04	0.12	0.36	0.970	Excluded because of proportion missing ≥ 95%
Infection	Hepatitis B virus c antibody;P	NPU02346	mean	0.059	0.236	0.00	0.00	0.00	0.971	Excluded because of proportion missing ≥ 95%
			min	0.059	0.236	0.00	0.00	0.00	0.971	Excluded because of

										proportion missing ≥ 95%
			max	0.059	0.236	0.00	0.00	0.00	0.971	Excluded because of proportion missing ≥ 95%
Organ markers	Carbamide;P	NPU01459	mean	5.119	1.723	4.10	4.90	5.80	0.973	Excluded because of proportion missing ≥ 95%
			min	5.055	1.729	4.10	4.80	5.80	0.973	Excluded because of proportion missing ≥ 95%
			max	5.182	1.748	4.20	4.90	5.90	0.973	Excluded because of proportion missing ≥ 95%
Organ markers	Brain natriuretic peptide;P	NPU17181	mean	10.006	18.910	2.50	5.10	10.20	0.975	Excluded because of proportion missing ≥ 95%
			min	9.250	18.250	2.30	4.80	9.38	0.975	Excluded because of proportion missing ≥ 95%
			max	10.936	20.555	2.60	5.35	10.78	0.975	Excluded because of proportion missing ≥ 95%
Allergy	Egg white antibody (f1) (IgE);P	NPU10996	mean	0.480	2.698	0.02	0.13	0.34	0.975	Excluded because of proportion missing ≥ 95%
			min	0.442	2.600	0.02	0.12	0.34	0.975	Excluded because of proportion missing ≥ 95%

			max	0.560	3.731	0.02	0.13	0.34	0.975	Excluded because of proportion missing ≥ 95%
Allergy	Peanut antibody (f13) (IgE);P	NPU11009	mean	1.970	9.259	0.02	0.17	0.53	0.976	Excluded because of proportion missing ≥ 95%
			min	1.892	9.164	0.02	0.16	0.50	0.976	Excluded because of proportion missing ≥ 95%
			max	2.073	9.659	0.02	0.17	0.54	0.976	Excluded because of proportion missing ≥ 95%
Allergy	Grain antibody (f4;7;8;10;11) (IgE);P	NPU26714	mean	0.393	2.289	0.03	0.17	0.34	0.977	Excluded because of proportion missing ≥ 95%
			min	0.387	2.288	0.03	0.14	0.34	0.977	Excluded because of proportion missing ≥ 95%
			max	0.399	2.296	0.03	0.17	0.34	0.977	Excluded because of proportion missing ≥ 95%
Immunology and inflammation	Cyclic citrullinated peptide antibody (IgG);P	NPU19947	mean	3.954	24.926	0.50	0.90	1.80	0.978	Excluded because of proportion missing ≥ 95%
			min	3.827	24.547	0.50	0.80	1.80	0.978	Excluded because of proportion missing ≥ 95%
			max	4.092	25.547	0.50	0.90	1.90	0.978	Excluded because of

										proportion missing ≥ 95%
Metabolism	Cholesterol+ester. in LDL. patient fasting status unknown;P	NPU01568	mean	3.010	0.930	2.34	2.97	3.60	0.979	Excluded because of proportion missing ≥ 95%
			min	2.984	0.931	2.30	2.95	3.59	0.979	Excluded because of proportion missing ≥ 95%
			max	3.035	0.943	2.36	2.98	3.63	0.979	Excluded because of proportion missing ≥ 95%
Organ markers	Creatine kinase;P	NPU19656	mean	115.413	131.499	65.00	88.00	124.00	0.979	Excluded because of proportion missing ≥ 95%
			min	104.850	101.270	63.00	83.00	118.00	0.979	Excluded because of proportion missing ≥ 95%
			max	133.976	305.138	66.00	90.00	128.00	0.979	Excluded because of proportion missing ≥ 95%
Allergy	Cladosporium herbarum antibody (m2) (IgE);P	NPU10876	mean	0.416	1.351	0.02	0.34	0.34	0.980	Excluded because of proportion missing ≥ 95%
			min	0.393	1.121	0.02	0.34	0.34	0.980	Excluded because of proportion missing ≥ 95%
			max	0.441	1.809	0.02	0.34	0.34	0.980	Excluded because of proportion missing ≥ 95%

Endocrinology	Phosphate(inorganic);P	NPU03096	mean	1.450	0.236	1.30	1.49	1.62	0.981	Excluded because of proportion missing ≥ 95%
			min	1.442	0.240	1.29	1.47	1.62	0.981	Excluded because of proportion missing ≥ 95%
			max	1.459	0.237	1.31	1.49	1.63	0.981	Excluded because of proportion missing ≥ 95%
Allergy	Soybean antibody (f14) (IgE);P	NPU11187	mean	0.568	3.591	0.01	0.11	0.34	0.981	Excluded because of proportion missing ≥ 95%
			min	0.544	3.575	0.01	0.11	0.34	0.981	Excluded because of proportion missing ≥ 95%
			max	0.600	3.650	0.01	0.12	0.34	0.981	Excluded because of proportion missing ≥ 95%
Allergy	Tomato antibody (f25) (IgE);P	NPU11214	mean	0.204	0.497	0.00	0.03	0.34	0.982	Excluded because of proportion missing ≥ 95%
			min	0.202	0.495	0.00	0.03	0.34	0.982	Excluded because of proportion missing ≥ 95%
			max	0.207	0.501	0.00	0.03	0.34	0.982	Excluded because of proportion missing ≥ 95%
Allergy	Meat antibody (f26;27;83) (IgE);P	NPU19618	mean	0.152	0.255	0.01	0.03	0.34	0.982	Excluded because of

										proportion missing ≥ 95%
			min	0.151	0.254	0.01	0.03	0.34	0.982	Excluded because of proportion missing ≥ 95%
			max	0.153	0.256	0.01	0.03	0.34	0.982	Excluded because of proportion missing ≥ 95%
Allergy	Hazel antibody (t4) (IgE);P	NPU10947	mean	2.570	9.709	0.00	0.02	0.34	0.983	Excluded because of proportion missing ≥ 95%
			min	2.537	9.675	0.00	0.02	0.34	0.983	Excluded because of proportion missing ≥ 95%
			max	2.604	9.762	0.00	0.02	0.34	0.983	Excluded because of proportion missing ≥ 95%
Allergy	Codfish antibody (f3) (IgE);P	NPU11215	mean	0.221	1.938	0.00	0.02	0.20	0.983	Excluded because of proportion missing ≥ 95%
			min	0.193	1.578	0.00	0.01	0.17	0.983	Excluded because of proportion missing ≥ 95%
			max	0.250	2.351	0.00	0.02	0.29	0.983	Excluded because of proportion missing ≥ 95%
Organ markers	Aspartate transaminase;P	NPU19654	mean	23.774	21.379	16.25	20.25	25.63	0.985	Excluded because of proportion missing ≥ 95%

	min	22.836	21.381	16.00	20.00	25.00	0.985	Excluded because of proportion missing ≥ 95%
	max	24.800	21.928	17.00	21.00	27.00	0.985	Excluded because of proportion missing ≥ 95%

Supplementary table S2: Overview of the of candidate predictors in the curated list

Analysis group	English Name	International Union of Pure and Applied Chemistry	Statistic property	Mean	Standard deviation	Q1	Median	Q3	Proportion missing	Note
Immunology and inflammation	Immunoglobulin A;P	NPU19795	mean	2.096	0.929	1.45	1.97	2.60	0.011	
			min	2.077	0.925	1.44	1.95	2.57	0.011	
			max	2.115	0.938	1.46	1.98	2.62	0.011	
Hematology	Erythrocyte mean corpuscular volume;Erc(B)	NPU01944	mean	88.783	5.419	86.00	89.00	92.00	0.084	
			min	87.617	5.526	85.00	88.00	91.00	0.084	
			max	89.996	5.812	87.00	90.00	94.00	0.084	
Hematology	Erythrocyte volumes; relative distribution width	NPU18162	mean	13.157	0.877	12.68	13.05	13.48	0.084	
			min	12.887	0.738	12.40	12.80	13.20	0.084	
			max	13.460	1.761	12.80	13.30	13.80	0.084	
Hematology	Haemoglobin;B	NPU02319	mean	8.401	0.741	7.90	8.33	8.88	0.084	
			min	8.201	0.816	7.70	8.20	8.70	0.084	
			max	8.597	0.754	8.10	8.50	9.10	0.084	
Organ marker	Alanine transaminase;P	NPU19651	mean	25.347	19.522	16.67	21.00	28.00	0.168	
			min	21.404	15.420	15.00	18.00	24.00	0.168	
			max	31.070	37.999	18.00	23.00	33.00	0.168	
Immunology and inflammation	C-reactive protein;P	NPU19748	mean	6.500	8.648	4.00	4.00	5.00	0.190	
			min	5.204	6.677	4.00	4.00	5.00	0.190	
			max	8.920	16.729	4.00	4.00	5.00	0.190	
Organ marker	Alkaline phosphatase;P	NPU19655	mean	38.019	29.394	22.73	28.33	36.67	0.214	
			min	35.791	28.700	21.25	26.25	34.17	0.214	
			max	40.582	32.085	23.75	30.00	40.00	0.214	

Tracers and vitamins	25-Hydroxy-Vitamin D(D3+D2);P	NPU10267	mean	61.930	25.106	45.00	59.80	76.27	0.393
			min	53.324	26.868	33.00	50.60	69.30	0.393
			max	71.064	30.834	51.00	68.20	88.00	0.393
Hematology	Cobalamin;P	NPU01700	mean	318.458	136.044	230.00	290.00	370.00	0.552
			min	301.295	130.338	220.00	275.00	355.00	0.552
			max	338.881	166.864	240.00	300.00	390.00	0.552
Hematology	Haemoglobin, MCHC;Erc(B)	NPU02321	mean	20.739	0.679	20.35	20.80	21.20	0.651
			min	20.608	0.764	20.20	20.70	21.10	0.651
			max	20.868	0.686	20.50	20.90	21.30	0.651
Hematology	Ferritin;P	NPU19763	mean	78.175	103.140	25.00	46.00	95.00	0.672
			min	73.048	98.388	22.00	42.00	90.00	0.672
			max	84.151	115.604	27.00	50.50	102.00	0.672
Hematology	Iron;P	NPU02508	mean	16.167	6.727	12.00	16.00	20.00	0.785
			min	15.484	6.953	11.00	15.00	19.00	0.785
			max	16.916	7.195	12.00	16.00	21.00	0.785
Hematology	Transferrin;P	NPU03607	mean	33.728	5.832	30.00	33.00	37.00	0.820
			min	33.378	5.809	29.00	33.00	36.00	0.820
			max	34.089	6.070	30.00	33.00	37.00	0.820
Hematology	Transferrin(Iron binding sites;Plasma)—Iron; substance fraction = ?	NPU04191	mean	0.250	0.114	0.17	0.24	0.31	0.830
			min	0.241	0.117	0.16	0.23	0.31	0.830
			max	0.259	0.119	0.18	0.25	0.33	0.830
Hematology	Folate;P	NPU02070	mean	20.457	11.943	11.96	17.20	25.70	0.923
			min	19.614	11.919	11.30	16.40	24.60	0.923
			max	21.363	12.756	12.20	17.80	26.78	0.923
Hematology	Reticulcyte, haemoglobin;Rtcs(B)	NPU17007	mean	1.871	0.201	1.78	1.92	2.00	0.937

			min	1.855	0.215	1.76	1.91	2.00	0.937	
			max	1.887	0.197	1.81	1.93	2.01	0.937	
Electrolytes	Calcium-ion free (pH=7,4);P	NPU04144	mean	1.232	0.039	1.21	1.23	1.26	0.953	Excluded because proportion missing ≥ 95%
			min	1.231	0.040	1.21	1.23	1.26	0.953	Excluded because proportion missing ≥ 95%
			max	1.233	0.040	1.21	1.23	1.26	0.953	Excluded because proportion missing ≥ 95%
Tracers and vitamins	Magnesium;P	NPU02647	mean	0.863	0.092	0.81	0.86	0.92	0.984	Excluded because proportion missing ≥ 95%
			min	0.857	0.102	0.80	0.86	0.92	0.984	Excluded because proportion missing ≥ 95%
			max	0.869	0.092	0.82	0.87	0.93	0.984	Excluded because proportion missing ≥ 95%
Metabolism	Methylmalonat;P	NPU02780	mean	0.254	0.470	0.15	0.18	0.24	0.989	Excluded because proportion missing ≥ 95%
			min	0.236	0.330	0.15	0.18	0.24	0.989	Excluded because proportion missing ≥ 95%

			max	0.267	0.599	0.15	0.19	0.25	0.989	Excluded because proportion missing ≥ 95%
Tracers and vitamins	Zink;P	NPU03768	mean	10.703	1.926	10.00	11.00	12.00	0.991	Excluded because proportion missing ≥ 95%
			min	10.582	2.050	9.00	11.00	12.00	0.991	Excluded because proportion missing ≥ 95%
			max	10.825	1.957	10.00	11.00	12.00	0.991	Excluded because proportion missing ≥ 95%
Hematology	Erythrocytes, vol.fr.;B	NPU01961	mean	0.405	0.035	0.38	0.41	0.43	0.993	Excluded because proportion missing ≥ 95%
			min	0.403	0.036	0.38	0.40	0.42	0.993	Excluded because proportion missing ≥ 95%
			max	0.407	0.036	0.38	0.41	0.43	0.993	Excluded because proportion missing ≥ 95%

Supplementary figure S3: Scatterplot showing the correlation between proportion missing and importance of predictor in the full model

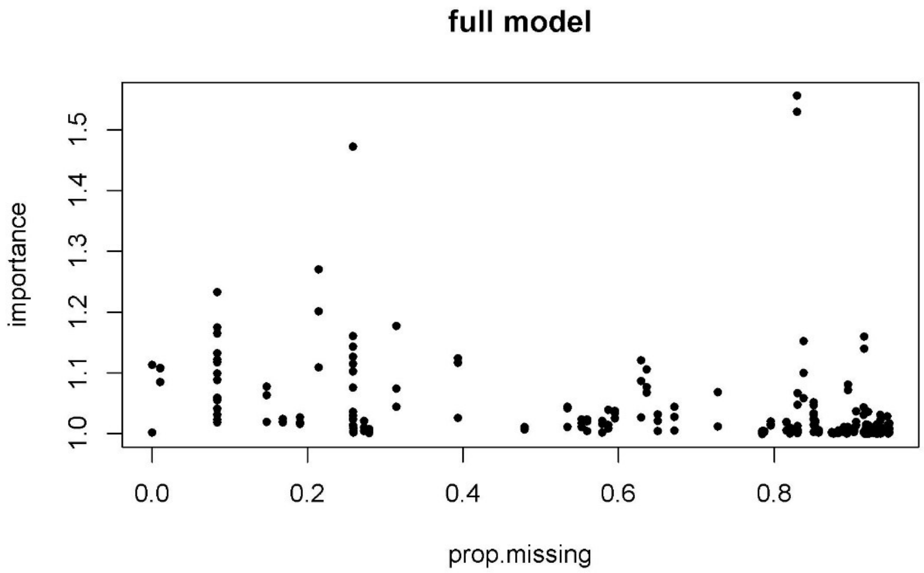

Supplementary figure S4: Scatterplot showing the correlation between proportion missing and importance of predictor in the curated model

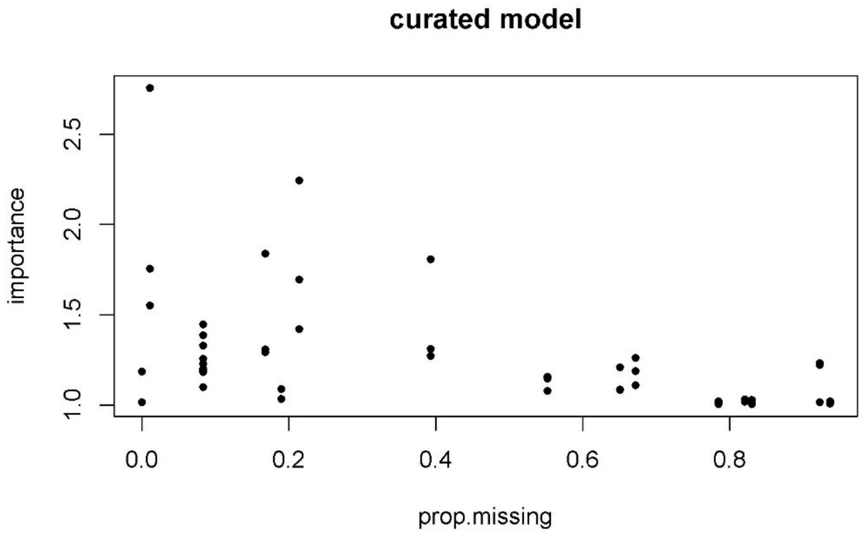

Supplementary figure S5: Decision curve analysis for the full model

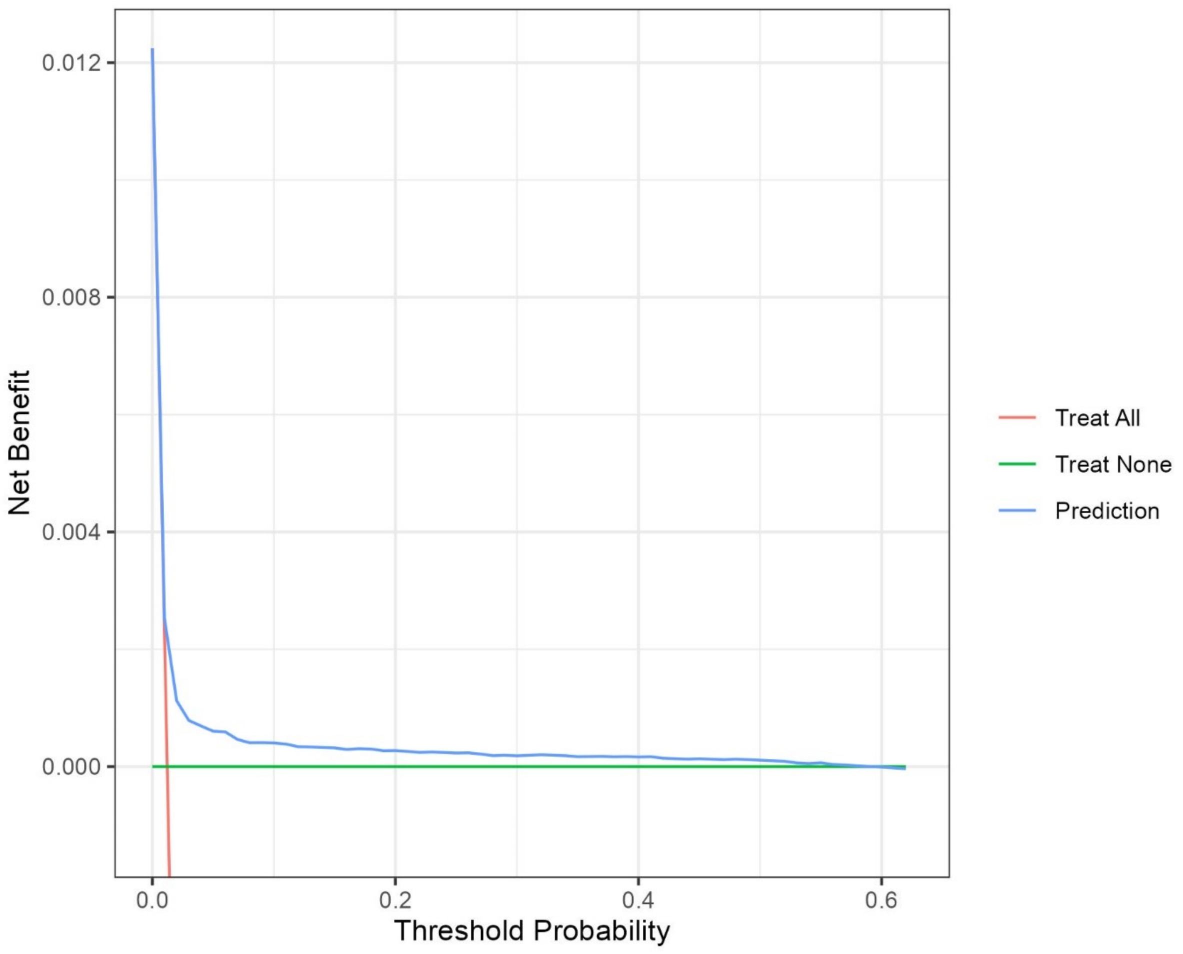

Supplementary figure S6: Area under the curves shown for subdivided look-back time in years for the full model

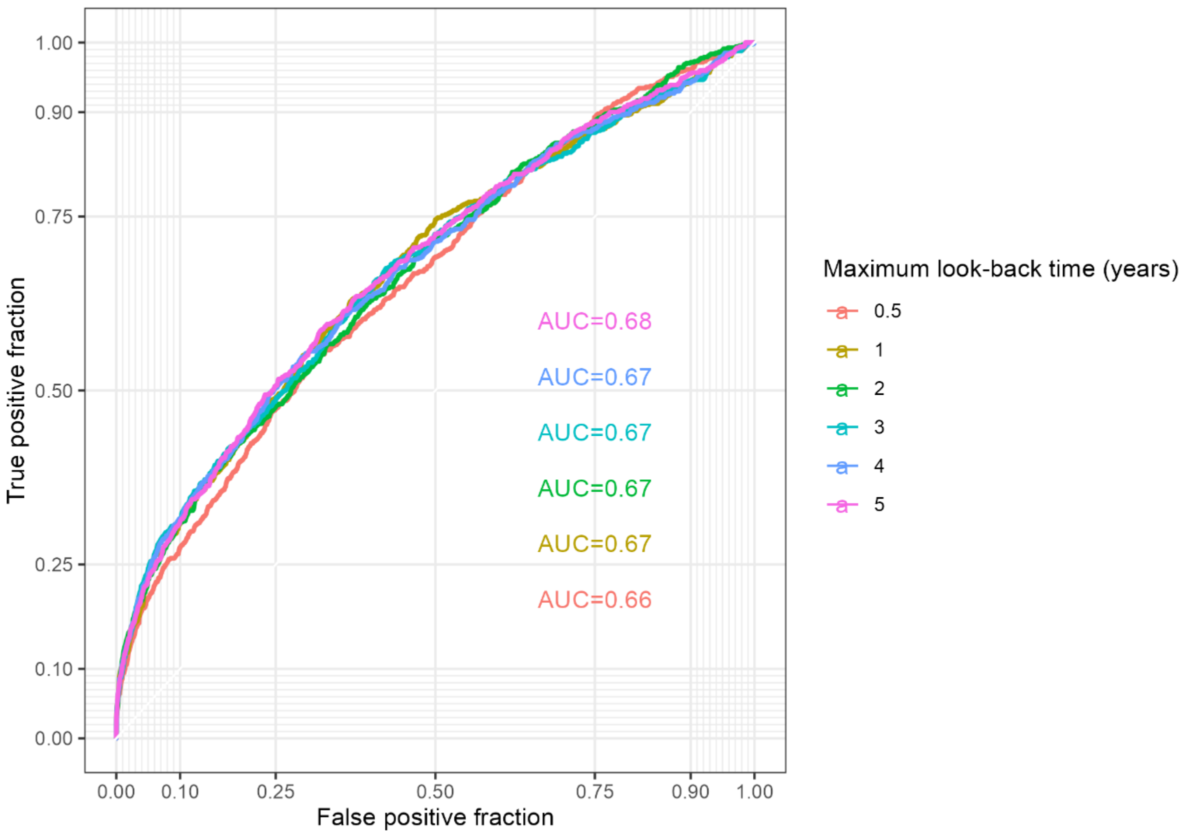

Supplementary Table S7: AUCs for the individual models

Name of model	AUC	Superlearner coefficient curated model	Superlearner coefficient full model
Curated model			
Extreme gradient boost	0.626	4.321	3.942
Forwards stepwise variable	0.635	0.731	0.06
Support vector machines	0.547	0.089	0
Lasso logistic penalized regression	0.662	6.108	2.907
Full model			
Extreme gradient boost	0.66	0	0.004
Forwards stepwise variable	0.68	0	2.736
Support vector machines	0.547	0	0.124
Lasso logistic penalized regression	0.688	0	6.132

Supplementary Table S8: 20 features with largest SHAP values in magnitude for the full model

Feature	Mean SHAP	2.5%	97.5%
Max food allergen antibody	0.0113641054876325	0.00167624259674007	0.0650235035904105
Mean neutrophilocytes	0.0108958949014387	0.000211560996956233	0.046089383039661
Mean food allergen antibody	0.0105134696068286	0.000122552524370472	0.0962558047104958
Mean alkaline phosphatase	0.0097993885795852	0.000402139014218763	0.0270732966962359
Max cholesterolester	0.00850505779283707	0.0043136432707177	0.0290739271591662
Max leukocytes	0.00754908919689889	7.49051355259647e-05	0.0461453645917811
Mean birch antibody	0.0073412111933005	0.000682007204654217	0.047614992113618
Max alkaline phosphatase	0.00695869926517953	0.000103712505778242	0.0193579619408962
Min thrombocytes	0.00606306596283158	8.5219704872728e-05	0.0517409466165157
Mean glomerular filtration	0.00539893330900293	0.000113190448588006	0.0345705414766792
Age at CD	0.00510980950794327	8.46477385578852e-05	0.0212650782871118
Max thrombocytes	0.00504071138864803	0.000397764180044558	0.0227967847794532
Min triglyceride fasting patient	0.00502499208793844	0.00141173281586812	0.0170600584188571
Min immunoglobin A	0.00484219936201415	0.000227071671264856	0.0178586552187934
Min alkaline phosphatase	0.00478764791514558	0.000197359502384547	0.0174542435216713
Max neutrophilocytes	0.00449301279202299	9.74939584623304e-05	0.0264717450360248
Mean eosinophilocytes	0.00448881719277691	7.70543179898307e-05	0.0195439009958683
Mean 25-hydroxy-vitamin	0.00437194511088684	9.86157191008558e-05	0.0272049908238677
Mean erythrocyte volumes	0.00423320130496929	8.79431981336824e-05	0.0221211604267616
Mean cholesterolester	0.00411449194399776	0.00087268497582563	0.0103607047086836
